# Treatment with gefitinib after erlotinib-induced liver injury: a case report

**DOI:** 10.1186/1752-1947-5-593

**Published:** 2011-12-21

**Authors:** Katsumi Nakatomi, Yoichi Nakamura, Iida Tetsuya, Shigeru Kohno

**Affiliations:** 1 Second Department of Internal Medicine, Nagasaki University School of Medicine, Nagasaki, Japan

## Abstract

**Introduction:**

Gefitinib and erlotinib have minor differences in their chemical structures, and thus it remains unclear whether the hepatotoxicity induced by one compound is affected by the other. The case of a patient who developed erlotinib-induced liver injury and was then treated with gefitinib without hepatic toxicity or disease progression is presented.

**Case presentation:**

A 31-year-old Japanese woman, who never smoked and who was diagnosed as having lung adenocarcinoma with carcinomatous meningitis, was treated with erlotinib. She developed erlotinib-induced liver injury after four weeks of treatment. The treatment was stopped right away, but the symptoms of meningitis re-appeared immediately. Gefitinib treatment was started and continued without recurrence of drug-induced liver injury.

**Conclusion:**

Gefitinib appears to be a potential treatment option after erlotinib-induced liver injury.

## Introduction

Gefitinib and erlotinib were the first oral epidermal growth factor receptor tyrosine kinase inhibitors (EGFR-TKIs) to become available in clinical practice. They have come to be very important drugs for patients with non-small cell lung cancer (NSCLC) harboring sensitive mutations of EGFR [1,2]. They have minor differences in their chemical structures, and thus it remains unclear whether the hepatotoxicity induced by one compound is affected by the other. The case of a patient who developed erlotinib-induced liver injury and was then treated with gefitinib without hepatic toxicity or disease progression is presented.

## Case presentation

**Figure 1:**
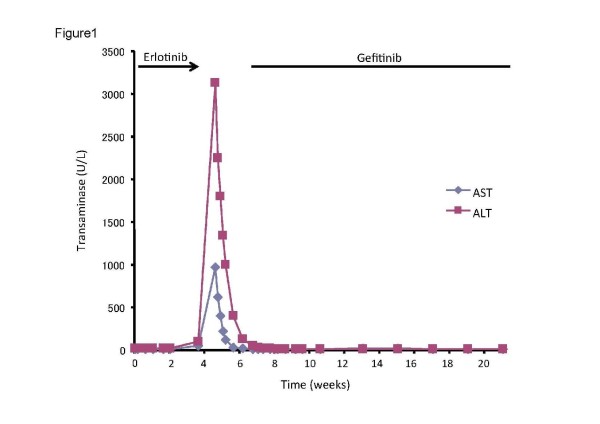
**Levels of aspartate aminotransferase (AST) and alanine aminotransferase (ALT) during erlotinib therapy and subsequent gefitinib therapy in a 31-year-old Japanese woman with non-small cell lung cancer (NSCLC)**.

A 31-year-old Japanese woman who never smoked presented with severe headache and nausea, and a performance status of approximately three. Gadolinium-enhanced magnetic resonance imaging (MRI) of her brain confirmed multiple metastatic brain tumors and meningitis, and a computed tomography (CT) scan of the chest confirmed a solitary, spiculated lesion in the left upper lung lobe. She had normal liver and renal functions, and she had no history of liver disease, excessive alcohol intake, drug abuse, or hepatitis. Cytology of the transbronchial brushings of the left upper lobe and the cerebrospinal fluid obtained by lumbar puncture revealed adenocarcinoma, and she was diagnosed as having a primary NSCLC with multiple brain metastases and carcinomatous meningitis. EGFR gene mutation analysis did not show the presence of any mutation. She was started on erlotinib 150 mg orally once daily because she refused to receive the emetic regimen. Two weeks after starting treatment, her headache and nausea had disappeared, and an MRI revealed brain tumor shrinkage and improvement in the meningitis. However, about four weeks after starting erlotinib, her transaminase levels started to increase, peaking at 972 U/L for aspartate aminotransferase (AST) and 3,130 U/L for alanine aminotransferase (ALT) (Figure 1). The patient had not complained of any clinical symptoms such as fever, nausea, diarrhea, abdominal pain, or fatigue, and she had taken no other medications or supplements. Serologic testing for hepatitis B and C was negative. An abdominal MRI revealed a normal liver with no other substantial abnormalities. Thus, erlotinib-induced liver injury was suspected and treatment was discontinued. After stopping erlotinib, her transaminase levels began to normalize at once, but severe nausea and headache also recurred immediately. Therefore, gefitinib 250 mg once daily was started with careful monitoring of liver function. Her nausea and headache improved quickly, and she continued to take gefitinib for more than 15 weeks without hepatic toxicity or disease progression. She was lost to follow-up, and she died of her cancer at another hospital.

## Discussion

**Figure 2:**
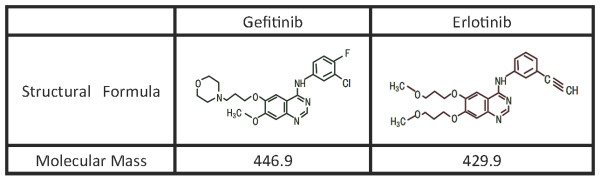
**Chemical structures of gefitinib and erlotinib**.

Erlotinib and gefitinib are orally bioavailable, synthetic anilinoquinazolines that selectively and reversibly bind to the intracellular ATP-binding site of EGFR tyrosine kinase (Figure 2) [1,2]. The hepatotoxicity of both drugs is usually mild to moderate and reversible on cessation of the drug. In Japan, Inoue *et al. *reported that, in a survey of 3000 NSCLC patients treated with gefitinib, 1.8% of patients developed grade 3 or 4 liver dysfunction or damage (with elevation of hepatic enzymes) [3]. Additionally, a prospective analysis of a gefitinib investigation reported that 11.1% of 3322 patients had suffered all-grade liver injury, and most of them were mild [4].

Erlotinib and gefitinib are both based on a 4-anilinoquinazoline kinase pharmacophore and exhibit similar pharmacokinetic characteristics in humans after oral administration, with extensive metabolism primarily by cytochrome P450 (CYP) 3A4 in liver; hepatotoxicities were common but mild in many cases [3-6]. In our patient, we were concerned about the possibility of drug-induced liver injury recurring while on gefitinib. It is well known that re-challenge or re-administration of the suspect drug following possible drug-induced liver injury can result in death or serious liver injury within a month [7]. The US Food and Drug Administration (FDA) recommends that re-challenge of subjects with significant (over 5 times the normal upper limit) transaminase elevations should not be attempted [8]. However, gefitinib did not result in the appearance of drug-induced liver injury in our patient. There have been similar reports regarding erlotinib treatment following gefitinib-induced liver injury [9,10]. Ku *et al. *hypothesized that, while the pharmacokinetics and basic chemical structures of both compounds are similar, minor differences in the chemical structure may account for differences in hepatotoxicity. They concluded that, in patients who develop gefitinib-induced hepatotoxicity but who are otherwise continuing to experience clinical benefit, consideration can be given to transitioning these patients to erlotinib [10]. In our patient, the opposite situation occurred, in that she developed liver toxicity on erlotinib and was successfully transitioned to gefitinib. Li *et al. *have examined the enzyme kinetics of gefitinib and erlotinib metabolism by individual CYP enzymes [11]. They found that gefitinib is more susceptible to CYP3A-mediated metabolism than erlotinib, and CYP2D6 was involved in gefitinib metabolism to a large extent, whereas CYP1A2 was extensively involved in erlotinib metabolism. These different metabolizing enzyme profiles may affect the drug-induced liver injury seen with each drug.

## Conclusion

Erlotinib and gefitinib are very important drugs for patients with NSCLC harboring sensitive mutations of EGFR. Following discontinuation of an EGFR-TKI due to hepatitis, the benefits and risks of other EGFR-TKI treatments should be considered. If the potential benefits outweigh the possible risks, and with appropriate monitoring, evaluating the safety of another EGFR-TKI may be warranted.

## Consent

Written informed consent was obtained from the patient's next of kin for publication of this case report and any accompanying images. A copy of the written consent is available for review by the Editor-in-Chief of this journal.

## Competing interests

The authors declare that they have no competing interests.

## Authors' contributions

NK, NY, and IT were the treatment team involved in the case. NY and KS wrote and edited the manuscript. All authors read and approved the final manuscript.
